# Diffusional spread and confinement of newly exocytosed synaptic vesicle proteins

**DOI:** 10.1038/ncomms9392

**Published:** 2015-09-24

**Authors:** Niclas Gimber, Georgi Tadeus, Tanja Maritzen, Jan Schmoranzer, Volker Haucke

**Affiliations:** 1 Leibniz Institut für Molekulare Pharmakologie (FMP) & Freie Universität Berlin, Department of Molecular Pharmacology and Cell Biology, Robert-Roessle-Straße 10, 13125 Berlin, Germany; 2 Charite Universitätsmedizin, NeuroCure Cluster of Excellence, Virchowweg 6, 10117 Berlin, Germany; 3 Freie Universität Berlin, Institut für Chemie und Biochemie, Takustrasse 6, 14195 Berlin, Germany

## Abstract

Neurotransmission relies on the calcium-triggered exocytic fusion of non-peptide neurotransmitter-containing small synaptic vesicles (SVs) with the presynaptic membrane at active zones (AZs) followed by compensatory endocytic retrieval of SV membranes. Here, we study the diffusional fate of newly exocytosed SV proteins in hippocampal neurons by high-resolution time-lapse imaging. Newly exocytosed SV proteins rapidly disperse within the first seconds post fusion until confined within the presynaptic bouton. Rapid diffusional spread and confinement is followed by slow reclustering of SV proteins at the periactive endocytic zone. Confinement within the presynaptic bouton is mediated in part by SV protein association with the clathrin-based endocytic machinery to limit diffusional spread of newly exocytosed SV proteins. These data suggest that diffusion, and axonal escape of newly exocytosed vesicle proteins, are counteracted by the clathrin-based endocytic machinery together with a presynaptic diffusion barrier.

## Abstract (web summary)

Neurotransmission is mediated by synaptic vesicles (SVs) fusion with the plasma membrane near active zones. Here, Gimber *et al.* observe that rapid diffusional spread and confinement is followed by slow reclustering of SV proteins at the periactive endocytic zone through SV protein association with the clathrin-based machinery.

## Graphical abstract

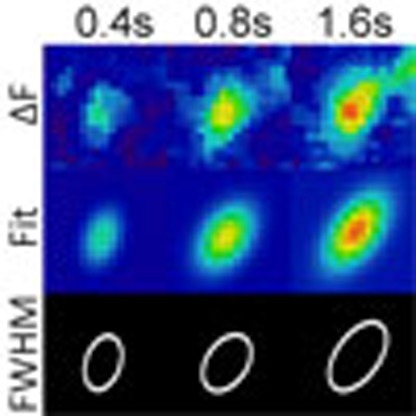

During synaptic activity non-peptide neurotransmitters are released by exocytic fusion of small clear SVs with the presynaptic membrane at active zones (AZs) followed by compensatory endocytosis of SV membranes from the surrounding periactive zone^1^,^2^,^3^,^4^,^5^. Although the effect of changes in surface mobility of postsynaptic neurotransmitter receptors on synaptic transmission is well established^6^,^7^,^8^,^9^, comparably little is known about the mobility of newly exocytosed SV proteins (NEP) within the presynaptic terminal. This is largely owed to the fact that SV exocytosis operates on a millisecond timescale and is immediately followed by endocytic membrane retrieval, thus, posing technical hurdles to the direct observation of the diffusional behaviour of newly exocytosed SV proteins in primary neurons. Plasma membrane pools of SV proteins (that is, originating from previous rounds of exocytosis) have been shown to either be dispersed within the axonal membrane^10^ or to cluster at presynaptic sites^11^. How these observations relate to the diffusional behaviour of newly exocytosed SV proteins remains unclear though. The fate of newly exocytosed SV proteins likely is of key importance for neurotransmission because the unlimited diffusional escape of SV proteins into the axon would counteract the reformation of properly sized SVs of correct composition by endocytosis. Interestingly, surface-stranded SV proteins have been shown to be preferentially retrieved by endocytosis to regenerate functional SVs^12^,^13^. Finally, diffusional dispersion of newly exocytosed SV proteins away from AZs may be crucial for the clearance of release sites during sustained high-level activity^14^,^15^. The diffusional behaviour of newly exocytosed membrane proteins has been assayed previously in fibroblasts^16^ and neuroendocrine PC12 cells^16^,^17^, which lack proper SVs and AZ-like release sites. Moreover, neuroendocrine cells frequently undergo kiss-and-run exocytosis^14^,^18^, thereby alleviating the need for a tight coupling between exocytic fusion and endocytic membrane retrieval that is characteristic of neurotransmission in central nervous system neurons^1^,^2^,^3^,^4^,^5^.

Here, we study the diffusional fate of newly exocytosed SV proteins in primary hippocampal neurons by high-resolution time-lapse imaging. We show that newly exocytosed SV proteins rapidly disperse within the first seconds post fusion until confined within the presynaptic bouton, followed by their slow reclustering. Confinement within the presynaptic bouton but not the rate of reclustering is modulated by SV protein association with the clathrin-based endocytic machinery to limit diffusional spread of newly exocytosed SV proteins. These data suggest that diffusion and axonal escape of newly exocytosed vesicle proteins are counteracted in part by SV protein association with the clathrin-based endocytic machinery and by the presence of a presynaptic diffusion barrier.

## Results

### Imaging newly exocytosed SV proteins

**Figure 1:**
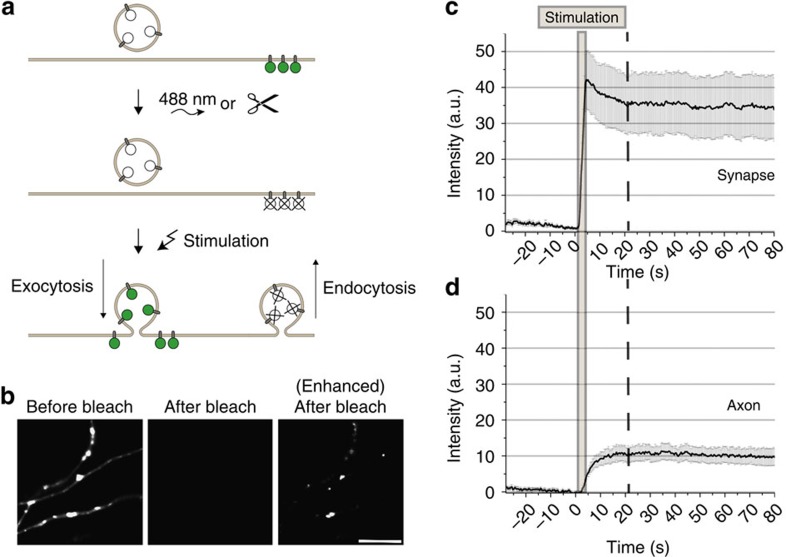
Newly exocytosed Syb2 is largely retained within presynaptic boutons. (**a**) Schematic of experimental workflow to monitor newly exocytosed pHluorin-tagged SV proteins. (**b**) Syb2-pHluorin fluorescence (F) before and after selective photobleaching. Right, enhanced contrast shows small residual F post-bleaching. Scale bar, 5 μm. (**c**,**d**) Syb2-pHluorin F intensity changes from time-lapse images (400 ms frame^−1^) of stimulated (40 APs, 20 Hz) surface-eclipsed neurons. (**c**) Presynaptic boutons, (**d**) axonal areas (mean±s.e.m.; *N*=10 neurons).

We expressed SV proteins lumenally tagged with pH-sensitive pHluorin GFP^19^,^20^ in cultured primary hippocampal neurons^10^,^13^. In resting neurons, the pHluorin signal is quenched because of the low intralumenal pH (5.5) of SVs, but is dequenched during stimulation-induced exocytosis to the neutral cell exterior^19^,^20^ (Fig. 1a, Supplementary Fig. 1a). During subsequent membrane retrieval and reacidification pHluorins are requenched (Supplementary Fig. 1a). To exclusively image newly exocytosed SV-pHluorin proteins we capitalized on the observation that SV proteins exocytosed and subsequently endocytosed are nonidentical^12^,^13^. Selective photobleaching or enzymatic digestion ‘eclipsed' the pre-existing surface pool of pHluorin-tagged synaptobrevin 2 (Syb2) (‘surface-eclipsed')^13^ (Fig. 1a,b). When prebleached neurons were stimulated with 40 action potentials (APs), we observed a fast initial rise in fluorescence (F) because of SV exocytosis, followed by a small decrease of F (by ∼15–20%) to a plateau level because newly exocytosed SV proteins were not endocytosed and reacidified^13^ (Fig. 1c). As a further confirmation we compared the Syb2-pHluorin responses of prebleached neurons, neurons treated with the vATPase inhibitor folimycin to prevent reacidification^21^, or prebleached folimycin-treated neurons. Under all of these conditions we observed a fast exocytic rise in F followed by a small decrease of F to a plateau level (Supplementary Fig. 1b), indicating that the large majority of all newly exocytosed proteins remains at the neuronal surface under these conditions rather than being reinternalized. The small F decrease at boutons observed post stimulation correlated with an F increase in the axon, suggesting that a minor fraction of newly exocytosed Syb2 escapes into the axon (Fig. 1d), possibly contributing to sharing of SV proteins between neighbouring boutons^22^. The majority (≥ 80%) of newly exocytosed Syb2, however, remained confined within the presynaptic bouton for up to 80 s (Fig. 1c).

**Figure 2:**
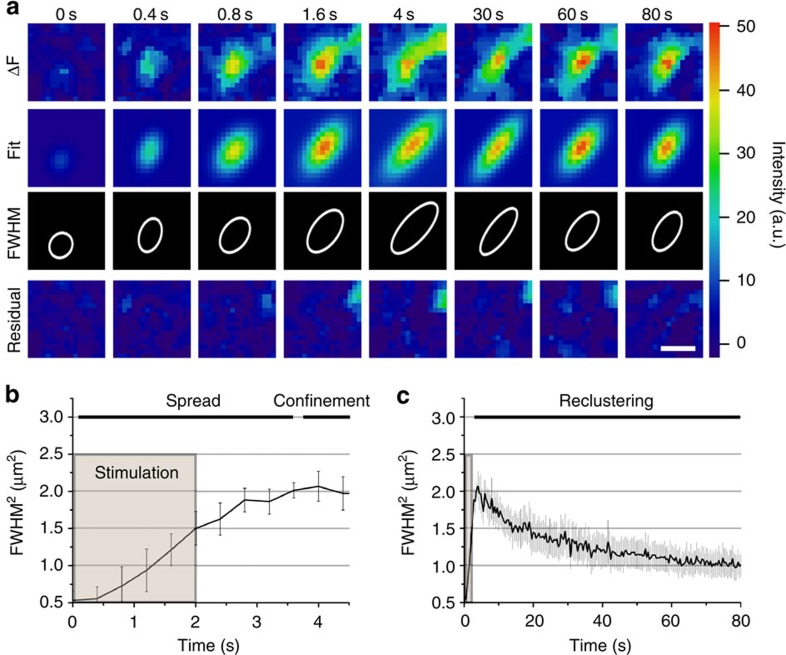
Syb2 spread, confinement and reclustering within presynaptic boutons. (**a**) Representative images of a Syb2-pHluorin expressing synapse of stimulated (40 APs, 20 Hz) surface-eclipsed neurons, either background subtracted (ΔF), Gaussian fit (Fit), FWHM (white line) or Gaussian fit residual. Scale bar, 1 μm. (**b**,**c**) FMWH^2^ of Gaussian fit over time. Fast diffusional spread during and after stimulation, followed by confinement (**b**) and reclustering (**c**). Mean±s.e.m.; *n*=5 independent experiments.

**Figure 3:**
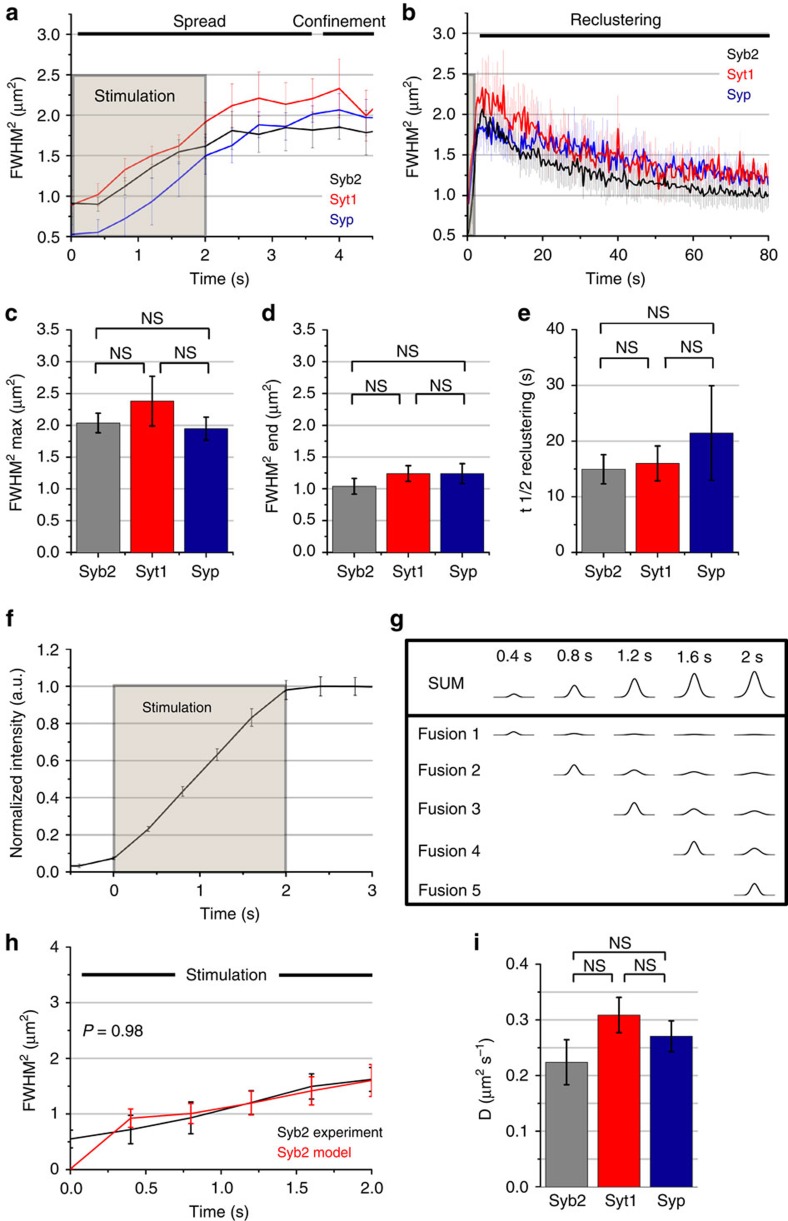
Diffusional spread, confinement, and reclustering of newly exocytosed SV proteins. (**a**,**b**) FMWH^2^ of Gaussian fit over time shows spreading, confinement (**a**), and reclustering (**b**) of Syb2-, Syt1- and Syp-pHluorin in surface-eclipsed stimulated (40 AP, 20 Hz) neurons imaged at 400 ms frame^−1^. (**c**–**e**) Maximal spread (**c**), last time point (**d**) and half-life t_1/2_ of reclustering (**e**). (**f**) Measured Syb2-pHluorin F increase used to model-free diffusion of successively fusing SVs. (**g**) Modelled intensity distributions from successive SV fusions and sum distribution. (**h**) Best-fit of model and experimentally determined FMWH^2^. (**i**) Diffusion coefficients (D) for Syb2, Syt1, Syp. Mean±s.e.m.; independent experiments: Syb2 (*n*=5), Syt1 (*n*=3), Syp (*n*=3). Statistical analysis was done by two-tailed unpaired t-test (c-e, i) or two-way analysis of variance with repeated measures (*h* time points 0.8–2 s). NS: *P*>0.05.

To image the precise spatiotemporal behaviour of newly exocytosed Syb2-pHluorin we combined high-resolution time-lapse imaging with synchronized electric field stimulation of surface-eclipsed neurons and semi-automated image analysis. Newly exocytosed pHluorin overlapped with the AZ marker Munc13-1 (Supplementary Fig. 1c) with 151 out of 160 boutons containing a single Munc13-1-positive AZ, suggesting that single synapses were imaged. Signals for newly exocytosed Syb2-pHluorin were then determined by fitting of the background- and bleach-corrected images (ΔF) to a two-dimensional (2D) Gaussian using a customized software tool (Fig. 2a). Excellent fits were obtained as demonstrated by the low, noise-like residuals (Fig. 2a, bottom). Hundreds of intensity and full-width half-maximum^2^ (FWHM^2^) traces recorded from surface-eclipsed neurons were averaged and quantitatively analysed (Fig. 2b,c). FWHM^2^ measured from the Gaussian fits of Syb2-pHluorin increased within the first few seconds of stimulation indicating fast local spread of newly exocytosed SV proteins (Fig. 2a,b), most likely corresponding to free diffusion (see Fig. 3 and below) within the bouton membrane. Diffusional spread within 2 s post stimulation reached a plateau indicative of the confinement of Syb2-pHluorin in an area of about 2 μm^2^, corresponding to the known dimensions of an average presynaptic bouton^23^. The short plateau phase was independent of variable expression levels of Syb2-pHluorin (Supplementary Fig. 1d) and was followed by a slow decrease of FWHM^2^ that continued for about 80 s until it reached about 1 μm^2^ (Fig. 2c). The observed decrease of FWHM^2^ was not due to photobleaching (Supplementary Fig. 1e), but rather reflected the time-dependent reclustering of exocytosed Syb2, consistent with results from stimulated emission depletion (STED) microscopy of fixed samples^11^. Moreover, diffusional spread, confinement, and reclustering of newly exocytosed SV proteins similar to that seen in photobleached neurons was observed in neurons expressing a tobacco etch virus (TEV)-cleavable variant of Syb2-pHluorin following TEV protease-mediated enzymatic digestion of the pre-existing surface pool of Syb2-pHluorin (Supplementary Fig. 1f–h).

### Spread, confinement and reclustering of major SV proteins

To probe whether diffusional spread, confinement and slow reclustering were specific properties of exocytosed Syb2 or reflect the general behaviour of all SV proteins we monitored the spatiotemporal dynamics of newly exocytosed pHluorin-tagged synaptophysin (Syp) and synaptotagmin 1 (Syt1), two other major SV proteins. Syt1 and Syp both exhibited diffusional spread within the first seconds of stimulation (Fig. 3a) until confined within the 2–2.5 μm^2^-sized area of the bouton (Fig. 3a,c), followed by their slow reclustering (Fig. 3b,d) with a *t*_1/2_ of 15–20 s (Fig. 3e). To test whether the initial spread of newly exocytosed SV proteins was mediated by free diffusion we fed the experimentally detected stimulation-induced increase in Syb2-pHluorin fluorescence (Fig. 3f) into a free diffusion model based on successively fusing SVs. Modelled intensity distributions from single events were summed (Fig. 3g), and the diffusion coefficient was adapted to yield the best-fit between model and experimentally determined FMWH^2^ for the initial SV protein spread during stimulation (Fig. 3h). This analysis revealed that the initial spread of newly exocytosed SV proteins indeed is described well by free diffusion within the first few seconds. We obtained similar diffusion coefficients for the initial dispersion of Syb2, Syt1 and Syp of ∼0.20–0.35 μm^2^ s^−1^ (Fig. 3i; Supplementary Fig. 2a,b), a value resembling that reported for mobile postsynaptic AMPA receptors^24^. This value would translate into a movement of SV proteins away from AZs at a speed of ∼1 μm in 2 s, explaining the need for confinement of newly exocytosed SV proteins within presynaptic boutons to prevent their substantial loss into axons.

### The endocytic machinery limits spread and confinement

**Figure 4:**
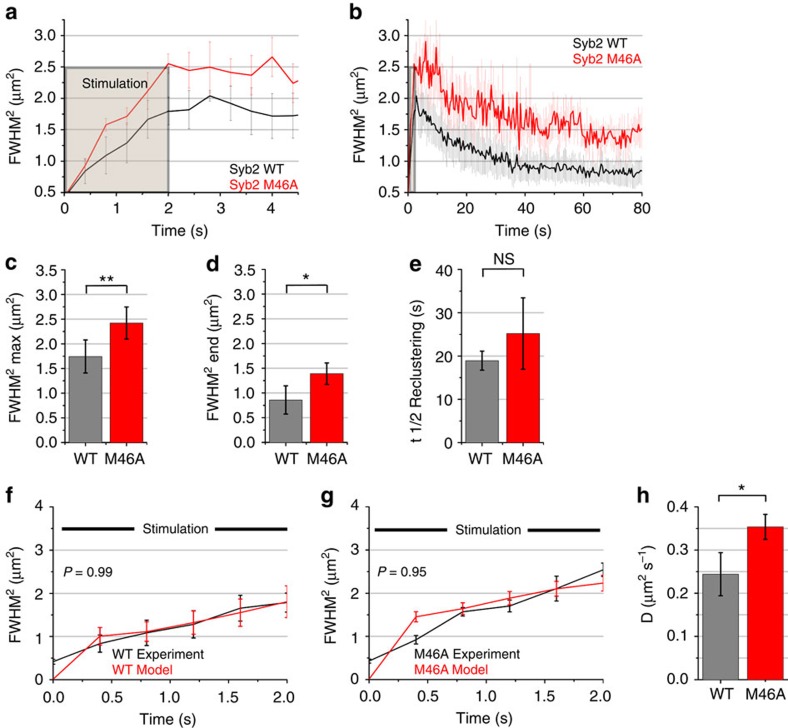
Spread and confinement of Syb2 are modulated by its association with AP180 or CALM. (**a**,**b**) FMWH^2^ of Gaussian fit over time shows spreading, confinement (**a**), and reclustering (**b**) of Syb2-pHluorin WT or M46A in surface-eclipsed stimulated (40 AP, 20 Hz) neurons imaged at 400 ms frame^−1^. (**c**–**e**) Maximal spread (**c**), last time point (**d**) and half-life *t*_1/2_ of reclustering (**e**). (**f**,**g**) Best-fit of model and experimentally determined FMWH^2^ for Syb2 WT and M46A. (**h**) Diffusion coefficients for Syb2 WT and M46A. Mean±s.e.m.; *n*=3 independent experiments. Statistical analysis was done by two-tailed paired *t*-test (**c**–**e**,**h**) or two-way analysis of variance with repeated measures (**f**,**g**) time points 0.8–2 s). **P<0.01, *P<0.05, NS: *P*>0.05.

As surface-stranded SV proteins form a reservoir for endocytic membrane retrieval^12^, we hypothesized that association of SV proteins with the endocytic machinery contributes to their presynaptic confinement. To test this, we analysed the post-exocytic diffusional behaviour of Syb2 (M46A), a missorted Syb2 mutant^25^ that selectively fails to associate with the Syb2-specific endocytic adaptors AP180 and CALM^26^,^27^ resulting in its partial redistribution (∼70% of total) to the plasma membrane^26^ (Supplementary Fig. 3a). Surface-eclipsing of the large surface-stranded pool of Syb2 (M46A)-pHluorin by enzymatic TEV protease-mediated digestion (Supplementary Fig. 3b) allowed us to nonetheless reliably track its diffusional fate, albeit with reduced signal-to-noise ratio. Syb2 (M46A) displayed rapid diffusional dispersion, confinement and slow reclustering similar to wild-type (WT) Syb2 (Fig. 4a,b). However, diffusing M46A explored a significantly larger area than WT Syb2 (Fig. 4a–c), and this difference in the FMWH^2^ remained after M46A had undergone slow reclustering (Fig. 4d), while *t*_1/2_ of reclustering was largely unaffected in the mutant (Fig. 4e). Furthermore, kinetic mathematical modelling of our experimental data revealed a significantly increased initial rate of diffusion of M46A compared with WT Syb2 (Fig. 4f–h).

**Figure 5:**
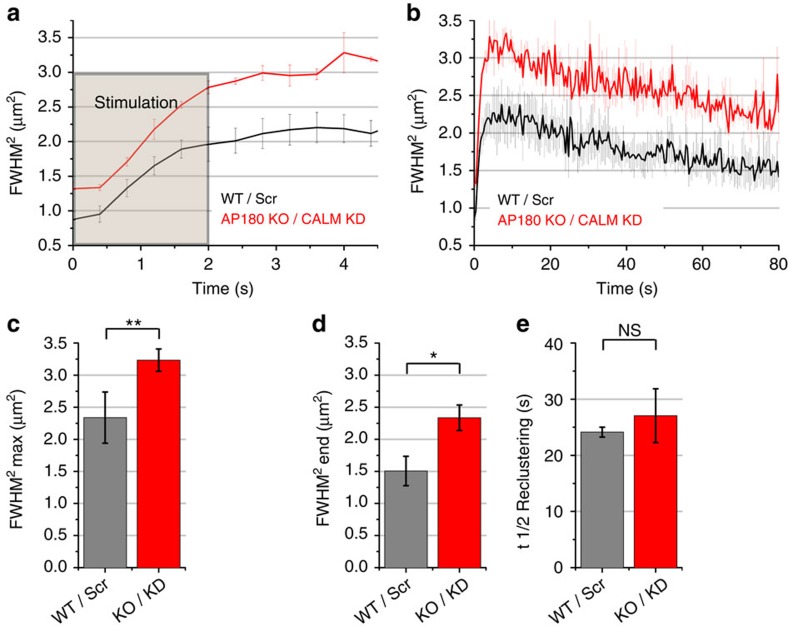
AP180 and CALM modulate spread and confinement of newly exocytosed Syb2. (**a**,**b**) FMWH^2^ of Gaussian fit over time shows spreading, confinement (**a**) and reclustering (**b**) of Syb2-pHluorin in surface-eclipsed hippocampal neurons lacking AP180 (KO) and depleted of CALM (knockdown (KD)) or corresponding WT control neurons treated with scrambled (Scr) shRNA (stimulation: 40 AP, 20 Hz). (**c**–**e**) Maximal spread (**c**), last time point (**d**) and half-life *t*_1/2_ of reclustering (**e**). Mean±s.e.m.; *n*=3 independent experiments). Statistical analysis was done by two-tailed paired *t*-test. **P<0.01, *P<0.05, NS: *P*>0.05.

To further investigate the contribution of the endocytic machinery to presynaptic confinement of newly exocytosed Syb2, we eliminated neuronal expression of the Syb2-specific endocytic adaptor proteins CALM and AP180 (refs 26, 27). When WT Syb2-pHluorin was expressed in neurons lacking AP180 (Supplementary Fig. 3c) and depleted of CALM by specific shRNA^28^ (Supplementary Fig. 3d), newly exocytosed pHluorin molecules explored a significantly larger area compared with WT neurons (Fig. 5a–c), and this difference in the FMWH^2^ remained after Syb2-pHluorin had undergone slow reclustering (Fig. 5d), while *t*_1/2_ of reclustering was largely unaffected (Fig. 5e), thus phenocopying the effects of Syb2 (M46A). We conclude that sustained abrogation of Syb2 association with its endocytic adaptors AP180 and CALM either by mutation of Syb2 (that is, M46A) or elimination of AP180/CALM expression modulates its diffusional spread and confinement. Consistent with these data we observed that acute perturbation of the endocytic machinery by the clathrin inhibitor Pitstop 2 (ref. 29; see also refs 30, 31, 32) also led to a similar increase in the area of Syb2 confinement (Supplementary Fig. 3e–h).

**Figure 6:**
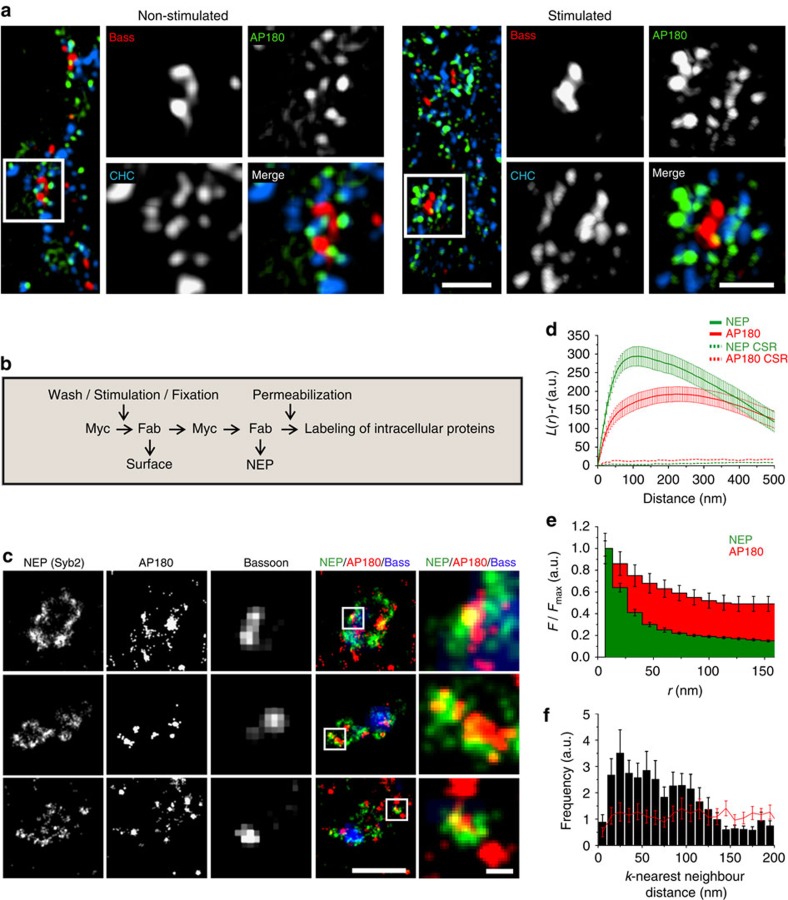
Newly exocytosed proteins co-cluster with endocytic proteins in the periactive zone. (**a**) 3D-Structured Illumination Microscopy (3D-SIM) analysis of Bassoon (Bass), AP180 and clathrin heavy chain (CHC) in stimulated (40 APs, 20 Hz) or non-stimulated hippocampal neurons. Scale bar, 1 μm; zoom, 500 nm. (**b**) Schematic for immunolabeling of the newly exocytosed SV protein pool (NEP). (**c**–**e**) SD-*d*STORM imaging of newly exocytosed SV proteins (stimulation: 40 AP, 20 Hz) in hippocampal neurons expressing Syb2-c-myc. (**c**) 2-colour SD-*d*STORM analysis of NEP and AP180, overlaid with wide-field image of Bassoon. Scale bar, 1 μm; zoom, 100 nm. (**d**) Ripley's *L*(*r*)-*r* function shows sub-synaptic clustering of NEP and of AP180, indicated by positive *L*(*r*)-*r* values with a maximum at radii of about 100 nm (NEP) and 200 nm (AP180). Mean±s.e.m.; *n*=31 synapses. Dotted lines represent upper envelopes of complete spatial randomness (CSR). (**e**) Radial intensity profiles of NEP and AP180 signals centred on the maxima of sub-synaptic NEP clusters show colocalization of both proteins (mean±s.e.m.; *n*=47 synapses). (**f**) *k*-Nearest neighbour analysis (k=10) of NEP and AP180 showing a maximum at 25 nm. The red line represents results after toroidal shift of one channel. Values above the red line indicate colocalization of NEP and AP180 (mean±s.e.m.; *n*=31 synapses).

Collectively, these data suggest that diffusional spread and confinement of newly exocytosed Syb2 are limited at least in part by its association with endocytic proteins. To investigate this hypothesis further we analysed the localization and dynamics of the clathrin-based endocytic machinery. Live imaging of hippocampal neurons co-expressing Munc13-1-mCherry or Syb2-pHluorin and fluorescent protein-tagged clathrin light chains (LC) showed clathrin to be concentrated in presynaptic boutons, where it localizes at and around AZs (Supplementary Fig. 4a,b), in close proximity to newly exocytosed SV proteins (Supplementary Fig. 4c,d). The distribution of eGFP-clathrin LC or mRFP-clathrin LC following stimulation of neurons with 40 APs remained grossly unchanged (Supplementary Fig. 4b,d). To more precisely determine the sub-synaptic localization of endocytic proteins and their spatial relationship with the pool of newly exocytosed SV proteins we turned to multicolour super-resolution microscopy. We first used three-dimensional (3D) Structured Illumination Microscopy (3D-SIM), a technique that provides an approximately 2-fold gain in both lateral (*xy*) and axial (*z*) resolution compared with conventional confocal imaging and allows the use of conventional fluorophores. Multicolour 3D-SIM analysis of hippocampal neurons revealed clathrin and AP180 to localize around the AZ labelled with Bassoon (Fig. 6a), and this distribution remained largely unaffected by stimulation of neurons with 40 APs, consistent with live imaging (Supplementary Fig. 4a–d). Finally, to determine the exact spatial relationship between the endocytic machinery and newly exocytosed SV proteins we used spectral demixing direct stochastic optical reconstruction microscopy (SD-*d*STORM)^33^, a dSTORM method that allows registration-free dual-colour imaging (Supplementary Fig. 4g) with a lateral resolution of 20–35 nm (refs 33, 34). Dual-colour SD-*d*STORM analysis of hippocampal synapses stimulated with 40 APs revealed newly exocytosed Syb2 molecules (Supplementary Fig. 4e,f) to cluster around the AZ labelled by Bassoon (Fig. 6c). Both, AP180 and newly exocytosed Syb2 formed sub-synaptic clusters (Fig. 6d, Supplementary Fig. 4h), which partially colocalized, indicated by an overlap of both maxima in the radial intensity profiles on sub-synaptic clusters (Fig. 6e) and by the peak in the *k*-nearest neighbour analysis (∼20–30 nm) that lies below the cluster size and within the range of the experimental resolution (Fig. 6f)^34^. The sub-synaptic NEP clusters had average radii of ∼50 nm (Fig. 6e), consistent with a distribution peak at distances of 100 nm (Fig. 6d). Interestingly, the surface area of these 100 nm flat clusters is similar to the surface area of native spherical SVs in brain with a diameter of ∼40–45 nm (ref. 35). This suggests that newly exocytosed SV protein clusters likely correspond to the readily retrievable pool of SV proteins, which following endocytosis gives rise to SVs. We conclude that confinement of SV proteins within presynaptic boutons is effected in part by their association with components of the endocytic machinery.

## Discussion

**Figure 7:**
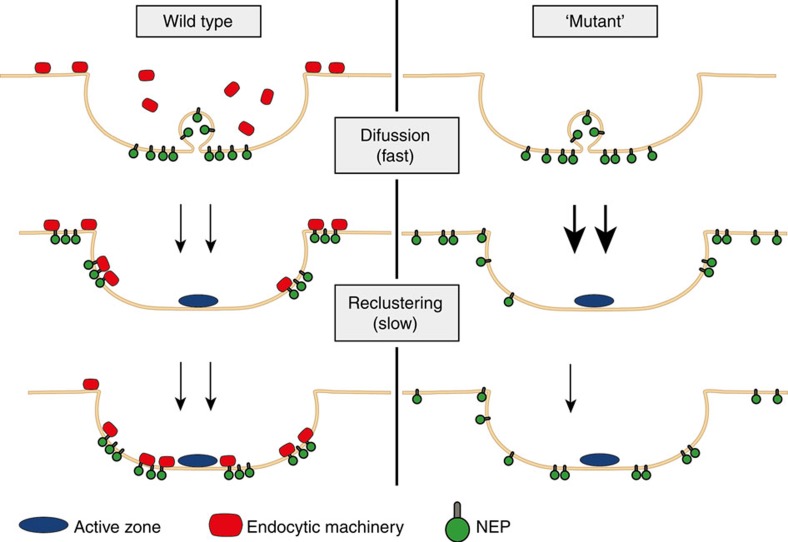
Model of protein diffusion, confinement and reclustering. Schematic model of SV protein confinement and reclustering and the role of the endocytic machinery: following rapid diffusion (WT, top) newly exocytosed SV proteins remain confined within the presynaptic bouton (WT, middle), aided by their association with the endocytic machinery. Confinement is followed by SV protein reclustering within the periactive zone (WT, bottom). In the absence of a functional endocytic machinery diffusion of newly exocytosed SV proteins is accelerated (bold arrows) and both confinement and the extent, but not the rate, of reclustering are compromised.

Our data based on high-resolution imaging and Gaussian fit analysis show that newly exocytosed SV proteins following an initial short phase of free diffusion are confined within presynaptic boutons, a process aided by their association with the endocytic machinery (Figs 4, 5, 6), in agreement with earlier studies in *Drosophila* employing an acute block of endocytosis in dynamin/shibire^ts^ mutants^36^. However, given the moderate effect of abolishing association of Syb2 with AP180 and CALM by mutation or by depleting neurons of both endocytic proteins, it is likely that escape of newly exocytosed SV proteins from the boutons into the axon is further limited by the presence of a presynaptic diffusion barrier, the identity of which remains unknown. We therefore suggest a model according to which newly exocytosed SV proteins diffusionally spread within the bouton, where they are captured by endocytic adaptors (e.g. AP180 and CALM for Syb2) localized around AZ release sites (Figs 6, 7)^37^,^38^. Endocytic SV protein association in this model, thus, not only aids sorting of SV proteins, but also restricts diffusional escape of newly exocytosed vesicle proteins into the surrounding axon. This model is also consistent with recent data from neuromuscular junctions, where the pool of actively recycling SVs has been shown to be kept largely separate from the reserve pool^39^ and with super-resolution imaging of the mesoscale organization of exocytic membrane proteins in neuroendocrine PC12 cells, where they form patches^40^ similar in size to newly exocytosed SV protein clusters observed here (see Fig. 6).

Diffusional spread and confinement of newly exocytosed SV proteins is incompatible with kiss-and-run SV exo-/endocytosis in central nervous system neurons, in which case exocytosed SV proteins would remain confined to the site of fusion for immediate retrieval^41^,^42^ without intermixing with plasma membrane components^10^. Similar observations have been reported for the exocytosis and rapid recapture of vesicular acetylcholine transporter in PC12 cells^17^, although in this case it has remained unclear to what extent recapture may reflect clathrin-mediated endocytosis of the transporter.

Following confinement, we observe slow reclustering of exocytosed SV proteins within the periactive zone. The extent but not the rate of reclustering depends on association of SV proteins with the endocytic machinery (Figs 4, 5), suggesting the involvement of additional factors. These may include interactions between SV proteins themselves^43^,^44^,^45^ and/or of SV proteins with membrane lipids such as cholesterol^46^, or components of the cytoskeleton. Interestingly, treatment of neurons with latrunculin A did not affect SV protein diffusion or reclustering in our hands (Supplementary Fig. 5). Hence, filamentous actin is unlikely to play an essential role in these processes, although it remains possible that local actin pools in presynaptic boutons are resistant to the drug or that short actin filaments or formin-dependent actin hotspots^47^ suffice to prevent SV protein diffusion and/or to facilitate reclustering. Such reclustered SV proteins likely constitute the readily retrievable pool of SVs^12^ that may subsequently be internalized by clathrin-dependent^48^ or bulk endocytosis mechanisms including ultrafast endocytosis^49^,^50^. We therefore propose that diffusional spread, confinement, and reclustering of SV proteins represent key steps in the SV exo-endocytic cycle that underlies neurotransmission.

## Methods

### Neuronal cell cultures and transfection

Primary hippocampal neuron cultures were prepared from mouse brain (P0-P3) and transfected at 7–8 days *in vitro* (DIV) by a modified calcium phosphate transfection protocol (Promega). Neurons were imaged at 14–15 DIV.

### Live cell imaging of hippocampal neurons

Images were acquired using an inverted fluorescence microscope (Eclipse Ti, Nikon), controlled by MicroManager 4.11 (ref. 51), equipped with a × 60 oil-immersion objective (*NA*=1.49, Nikon), a sCMOS camera (Neo, Andor) and a 200 Watt mercury lamp (Lumen 200, Prior). Images were acquired at 2.5, 0.5 or 0.2 Hz with the corresponding filter set (EGFP/pHluorin: F36-526, mCherry/mRFP: F36-504, AHF Analysentechnik). Imaging was performed at 30 °C in physiological imaging buffer (170 mM NaCl, 3.5 mM KCl, 5 mM NaHCO_3_, 5 mM glucose, 1.2 mM Na_2_SO_4_, 1.2 mM MgCl_2_, 1.3 mM CaCl_2_, 0.4 mM KH_2_PO_4_, 20 mM TES (2-[(2-Hydroxy-1,1-bis(hydroxymethyl)ethyl)amino]ethanesulfonic acid), pH 7.4). 50 μM APV (DL-2-Amino-5-phosphonopentanoic acid, Sigma-Aldrich) and 10 μM CNQX (6-Cyano-7-nitroquinoxaline-2,3-dione, Sigma-Aldrich) were added to the imaging buffer to silence spontaneous neurotransmission. Unquenched surface pHluorin was either selectively bleached by illuminating the neurons for 400 s prior to stimulation or the pHluorin tag was removed by 5 min enzymatic digestion with 4 μg ml^−1^ TEV protease in imaging buffer. Both procedures were sufficient to eclipse fluorescence signal from the surface pool of SV proteins such that any change in pHluorin signals after stimulation originated only from newly exocytosed protein, but not from pre-existing surface pools. Neurons were stimulated by electric field stimulation with 40 APs (at 20 Hz, 100 mA) in a stimulation chamber (RC-47FSLP, Warner Instruments). In Supplementary Fig. 1b reacidification was blocked by treating neurons for 1 min with 67 nM folimycin (in ethanol, 1 μl ml^−1^ imaging buffer). For experiments shown in Supplementary Fig. 3e–g, hippocampal neurons were treated with DMSO (0.67 μl ml^−1^ imaging buffer) and stimulation-induced pHluorin responses were recorded. Neurons were then treated for 2 min with 20 μM Pitstop 2 (in DMSO, 0.67 μl ml^−1^ imaging buffer) before stimulation to fill endocytic slots with SV cargo. Following a second round of surface-bleaching stimulation-induced pHluorin responses were recorded. For experiments shown in Supplementary Fig. 5a,b hippocampal neurons were treated for 5 min with 10 μM Latrunculin A (in DMSO, 1 μl ml^−1^ imaging buffer) or DMSO before stimulation. For the experiments shown in Supplementary Fig. 5c,d hippocampal neurons were transfected with LifeAct-eGFP. Images were taken before (DMSO, 1 μl ml^−1^ imaging buffer) and after 5 min of treatment with 10 μM Latrunculin A (in DMSO, 1 μl ml^−1^ imaging buffer).

### Image analysis

Identification of responding synapses: synaptic areas displaying stimulation-induced pHluorin responses were identified based on stimulus-evoked changes in local fluorescence signal (F) using a custom-written ImageJ macro^52^. The synaptic area was calculated from the differential image (*F*_after_−*F*_before_) based on a histogram-based threshold procedure.

Correction for photobleaching and background: Regions of interest containing responding synapses were extracted and intensity traces were fit with an exponential decay (Equation. 1) for each single pixel until the start of stimulation.

Exponential decay:


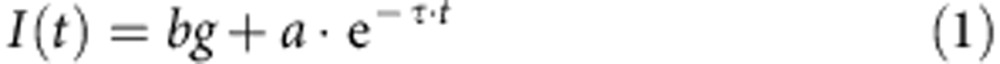


*I*(*t*): Intensity at time *t*; *bg*: background signal; *a*: intensity at time 0; *τ*: lifetime

The bleach rate was calculated from the lifetime of the pHluorin decay for each pixel (Equation. 2).

Bleach rate:


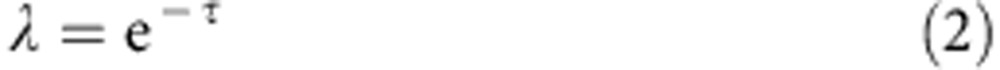


*λ*: bleach rate; *τ*: lifetime

The bleach rate was used to correct the pixel-specific bleaching over the entire image sequence (Equation. 3).

Bleach correction:


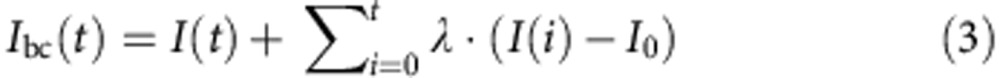


*I*_bc_ (*t*): bleach corrected intensity at time *t*; *I*(*t*): Intensity at time *t*; *: λ*: bleach rate; *I*_0_: intensity at time 0.

To reduce background and to correct for the residual fluorescence signal after photobleaching or TEV treatment (from partially quenched luminal pHluorin molecules), we subtracted the median of five images before stimulation from the entire image sequence resulting in ΔF (Fig. 2a).

Quantification of pHluorin intensity signal and escape: the pHluorin intensity signal over time was measured from selected (responding) synapses (see above). The escape of newly exocytosed SV proteins into the axon was quantified by manually selecting axonal regions of 0.9 μm^2^ with a distance of 1.2 μm to a responding synapse.

Quantification of local protein spread by Gaussian fit: The bleach and background corrected image sequences of responding synapses were fit frame-by-frame to a 2D Gaussian distribution using a custom-written Igor Pro macro (Equations 4, 5, 6). The maximum FWHM^2^ was analysed over time (Equation 7).

Gaussian fit 2D:


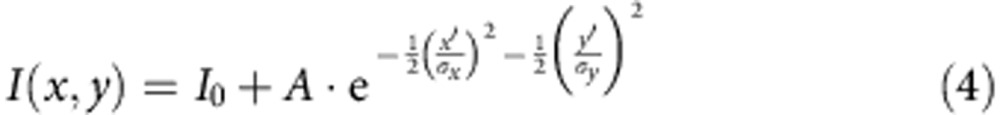



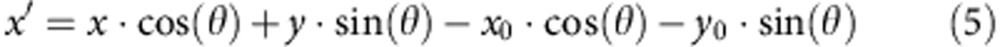



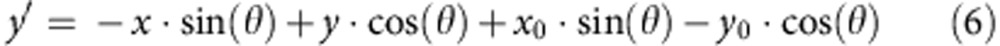



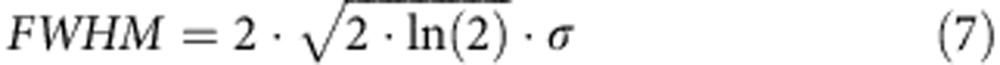


*I*(*x,y*): pixel intensity at the image coordinates *x*, *y*; *I*_0_: intensity offset; *A*: amplitude; *σ*: s.d.; *θ*: rotation angle; FWHM: full-width at half-maximum intensity; *x*_0_, *y*_0_: Gaussian centre.

Diffraction-limited rendering of SD-dSTORM images: to compare the SD-dSTORM images with images of conventional wide-field resolution the SD-*d*STORM images were blurred with a Gaussian in ImageJ using *σ*_Render_ from Equation 8.

Rendering:


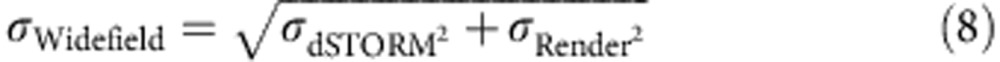


*σ*_Widefield_: standard deviation of wide-field image (Equations 7, 9); *σ*_dSTORM_: standard deviation of SD-dSTORM^34^; *σ*_Render_: standard deviation for rendering.

Wide-field resolution:


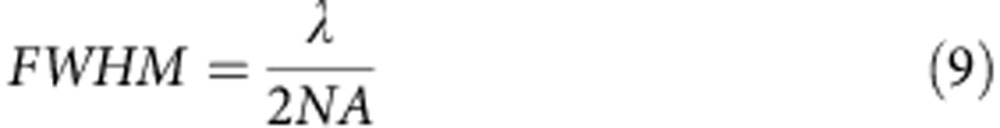


*FWHM*: full-width at half-maximum intensity; *λ*: emission wavelength of the dye; *NA*: numerical aperture of the objective

Finally, the blurred images were displayed with the camera pixel size of 105 nm.

Ripley's *L*(*r*)-*r* function (Fig. 6d): Estimation of normalized and edge-corrected Ripley's *K*(*r*) and *L*(*r*)-*r* function (Equations 10, 11) were calculated using the splancs package for the R software environment for statistical computing^53^ using the following equations^54^:

Ripley's:


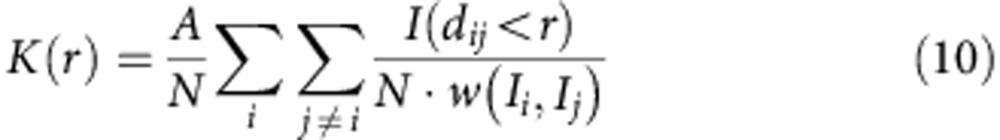



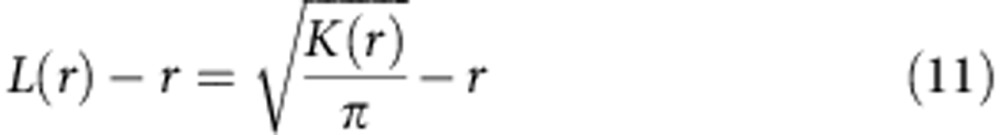


*K*(*r*): edge-corrected Ripley's *K* to estimate the number of individual localizations (*i*) within a distance r of another (*j*) localization normalized by the number of total localizations *N* per area *A*; *d*_*ij*_ is the distance between localization *i* and *j*; *I*(*d*_*ij*_<*r*) is the indicator function that equals 1 if *d*_*ij*_<*r* or 0 otherwise; *w*(*l*_*i*_*,l*_*j*_) is the weight function for edge correction.

*L*(*r*)-*r*: normalized *K*(*r*) equals 0 for a random distribution^55^. Dotted lines (Fig. 6d) represent upper envelopes from 10 simulations of complete spatial randomness (CSR) for each region of interest.

*K*-nearest neighbour: The *k*-nearest neighbour distribution was calculated from localizations within synaptic region of interests in SD-*d*STORM images, using a custom-written ImageJ macro^52^. *K*=10 was chosen in order to reduce the likelihood of false positive hits due to noise. The negative control for colocalization was generated by performing a toroidal shift of one channel for half a region of interest size against the other channel along the *x* axis.

Radial intensity profiles: Radial intensity profiles were generated with the ImageJ macro^52^ ‘radial profile'. Local maxima were found automatically with the ImageJ function ‘find maxima'. Averaged intensity values were plotted against their radial distance to a local maximum in the reference channel (newly exocytosed Syb2).

### Modelling diffusional spread of successive SV fusions

To determine diffusion coefficients of pHluorin-tagged synaptobrevin 2 (Syb2), synaptotagmin 1 (Syt1) and synaptophysin (Syp), we assumed a model of successively fusing SVs during stimulation. By using the experimentally determined intensity traces we calculated the relative number of fused SVs at each time point during stimulation. Assuming that the spread of the SV proteins is governed by Brownian motion (free diffusion) each vesicle was treated as an individual Gaussian intensity distribution that was added to the previous intensities. Each Gaussian distribution was weighted by its corresponding intensity increase (experimental data) and integrated with the previous Gaussian intensity distributions (Equations 12, 13). The sum of all distributions was fit with another Gaussian (Equation 14) and plotted as the ‘model' in Figs 3h,4f,g and Supplementary Fig. 2. The diffusion coefficients were determined by matching the mean slope from the experimental FWHM^2^ to the FWHM^2^ from the model. The validity of the model was tested by comparing traces obtained from the model and experimentally obtained traces, using a two-way analysis of variance with repeated measures.

Integrated Gaussian:


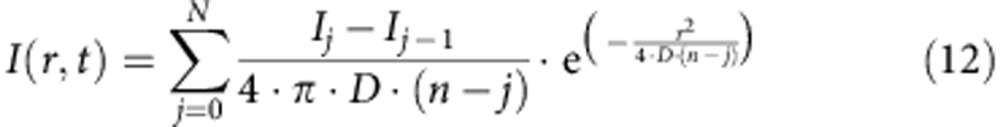



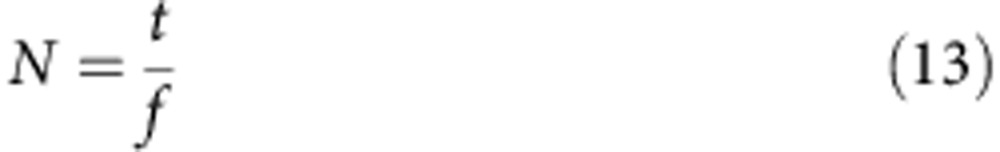


*I*(*r*, *t*): intensity at the radial distance *r* (from Gaussian center) and time *t*; *D*: diffusion coefficient; *n*: frame number; *f*: frame duration.

Gaussian Fit 1D:


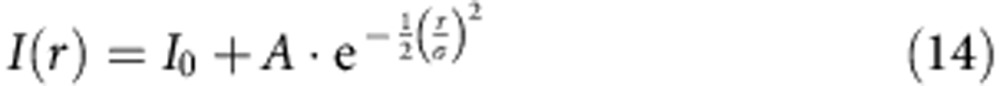


*I*(*r*): intensity at the radial distance *r* (from Gaussian centre); *I*_0_: intensity offset; *σ*: s.d.

### Immunocytochemistry

Primary hippocampal neurons were fixed with 4% (w/v) PFA, 4% (w/v) sucrose in phosphate-buffered saline for 15 min at room temperature (RT). Cells were permeabilized and blocked for 1 h, RT in blocking buffer (0.23% [v/v] Triton X-100, 385 mM NaCl in 15 mM sodium phosphate buffer pH 7.4, supplemented with 30% [v/v] goat serum or 10% [w/v] bovine serum albumin for staining with goat antibodies) before incubation with the specific primary antibody (1 h, RT) and the secondary antibody (30 min, RT) in blocking buffer.

### Immunocytochemistry of newly exocytosed proteins

Primary hippocampal neurons were transfected with c-myc epitope-tagged Syb2. On 14–15 DIV neurons were incubated with primary antibody in imaging buffer (20 min, RT). Neurons were washed with imaging buffer, stimulated in an electric field as described above and fixed with 4% (w/v) PFA, 4% (w/v) sucrose in phosphate-buffered saline for 15 min at RT. Further labelling steps were carried out in 15 mM sodium phosphate buffer (pH 7.4), supplemented with 30% (v/v) goat serum and 385 mM NaCl. Primary antibodies on the neuronal surface were saturated with Alexa 488-labelled Fab fragments of secondary antibody (overnight, RT), followed by a labelling of newly exocytosed proteins with primary (1 h, RT) and Alexa 647 labelled FAB fragments of secondary antibody (30 min, RT). Labelling of AP180 and Bassoon was performed with primary and secondary antibodies as described in the paragraph on immunocytochemistry.

### Image acquisition of fixed hippocampal neurons

For acquisition of images from immunostainings without the need for improved resolution were used the inverted fluorescence microscope setup, described above. To reduce out of focus fluorescence in neuronal cultures that also contained astrocytes (Supplementary Fig. 3d) we used a laser-scanning microscope LSM780 from (Carl Zeiss Microscopy GmbH) equipped with an Argon-Laser 488 and an DPSS-Laser. Emission was detected at 500–550 or 565–700 nm.

### Structured illumination microscopy

Hippocampal neurons were silenced for 5 min with 50 μM APV and 10 μM CNQX in imaging buffer, stimulated (40 APs, 20 Hz) or directly fixed and stained with primary and secondary antibodies as described before. Samples were mounted in Vectashield. 3D 3-colour SIM images were acquired using the 488, 568 and 643 nm laser lines, standard filter sets and 125 nm *z*-sectioning of the OMX V4 Blaze (GE Healthcare) system. 100 nm fluorescent beads (Tetraspek, T7284, Invitrogen) were used for registration of detection channels achieving <40 nm registration error for all three channels. Images were exported using Imaris 7.6 (Bitplane).

### SD-dSTORM

Spectral-demixing (SD)-*d*STORM provides registration error free, low crosstalk dual-colour super-resolution imaging with a resolution of ∼20–35 nm (refs 33, 34). The system, acquisition and analysis procedure was described previously^33^. Here, we used Alexa Fluor 647 (Invitrogen) and CF680 (Biotium) as dye pairs distinguishable by spectral demixing^34^. The emission splitter was equipped with a dichroic mirror (700-DCXXR, AHF Analysentechnik) and an emission bandpass filter (F76-635, AHF Analysentechnik). The overlay of SD-*d*STORM channels (NEP/AP180) with the wide-field channel (Bassoon) was done using a custom ImageJ macro^52^.

### Generation of AP180/CALM-depleted neurons

AP180 expression was eliminated by gene knockout (KO) in mice using homologous recombination: A floxed neomycin (NEO) resistance cassette was inserted in front of exon 3 and an additional loxP site after exon 6 in hybrid C57BL/6NTac x 129S6/SvEVTac ES cell clones injected into C57BL/6 blastocysts and implanted into pseudopregnant mice. Mating of chimeric males with Ella-Cre deleter mice removed exons 3–6 as well as the NEO cassette giving rise to heterozygous mice, which were interbred to obtain WT and AP180^**−/−**^ littermates. Animals were genotyped by PCR analysis of genomic DNA (see Supplementary Fig. 3c). The AP180 WT allele was detected with the forward primer ACCTCATGTGAAACGTTGCCTG and the reverse primer TCTGGTGGATAGTGTCACTTAGGTAG (product: 323 nt). The AP180 KO allele was detected using the forward primer CCAGATGACCTGAGTTTGTG and the reverse primer TCTGGTGGATAGTGTCACTTAGGTAG (product: 401 nt).

Primary hippocampal neuron cultures prepared from AP180^**−/−**^ mice (P0-P3) were transfected with pFUGW-based plasmids^56^ co-expressing mKate and specific shRNA directed against CALM^28^ to obtain AP180/CALM-depleted neurons. As a control, hippocampal neurons from WT mice (P0-P3) co-expressing mKate and scrambled control shRNA were used.

### Plasmids and antibodies

Plasmids encoding for Syb2 (WT, rat) and Syt1 (rat) with a TEV protease cleavable superecliptic pHluorin-tag were a kind gift from J. Klingauf (University of Münster, Münster, Germany)^7^. Mutant Syb2 (M46A) was cloned into a pHluorin plasmid by PCR amplification and standard cloning procedures. A PCR based approach was used to introduce a c-myc epitope tag into a plasmid encoding Syb2 (WT). The Syp-pHluorin plasmid (rat) was a kind gift from L. Lagnado (University of Sussex, UK)^48^. A plasmid encoding Munc13-1-mCherry (rat) was derived from pEGFP-N1 carrying Munc13-1, kindly provided by N. Brose (MPI for Experimental Medicine, Göttingen, Germany). Clathrin light chain (rat) was fused to mRFP and expressed from pcDNA5/FRT/TO and fused to eGFP, expressed from pcDNA3. The plasmid encoding for Lifeact-eGFP^57^ was a kind gift from F. Bradke (Center for Neurodegenerative Diseases, Germany). The CALM or scrambled shRNA was coexpressed with mKate from the pFUGW vector. The following antibodies were used: AP180 (155003, Synaptic Systems, 1: 100), Bassoon (141004, Synaptic Systems, 1: 500), CALM (sc-6433, Santa Cruz, 1: 100), clathrin heavy chain (ab21679, Abcam, 1: 400) c-myc epitope tag (9E10, purified from ascites, monoclonal, 1: 1,000).

### Statistical analysis

If not indicated differently, traces were recorded from 4–12 neurons per individual experiment. From each neuron several tens to hundreds of synapses were analysed. Data was pooled in two steps: (i) by calculating the median from all synapses from one neuron; and (ii) by using this value to generate the median for all neurons within each individual experiment. *N* individual experiments were averaged and used for statistical analysis. Maximal spread, FWHM^2^ of the last time point and the duration of half way reclustering were obtained from median filtered original traces in order to reduce noise. All values are depicted as mean±s.e.m. Statistical significance was calculated with Prism (Graphpad) using a two-way analysis of variance with repeated measures (for curves) or a two-tailed *t*-test (all bar graphs). Statistical significance is indicated as followed: **=*P*<0.01; *=*P*<0.05.

## Additional information

**How to cite this article:** Gimber, N. *et al.* Diffusional spread and confinement of newly exocytosed synaptic vesicle proteins. *Nat. Commun.* 6:8392 doi: 10.1038/ncomms9392 (2015).

## Supplementary Material

Supplementary Information Supplementary Figures 1-5
