# Supplementary material for: Diffusional spread and confinement of newly exocytosed synaptic vesicle proteins
Source: Nat Commun. 2015 Sep 24;6:8392. doi: 10.1038/ncomms9392 (PMC4598626; doi:10.1038/ncomms9392)
Supplement: Supplementary Information — Supplementary Figures 1-5 [file ncomms9392-s1.pdf]

Supplementary figures

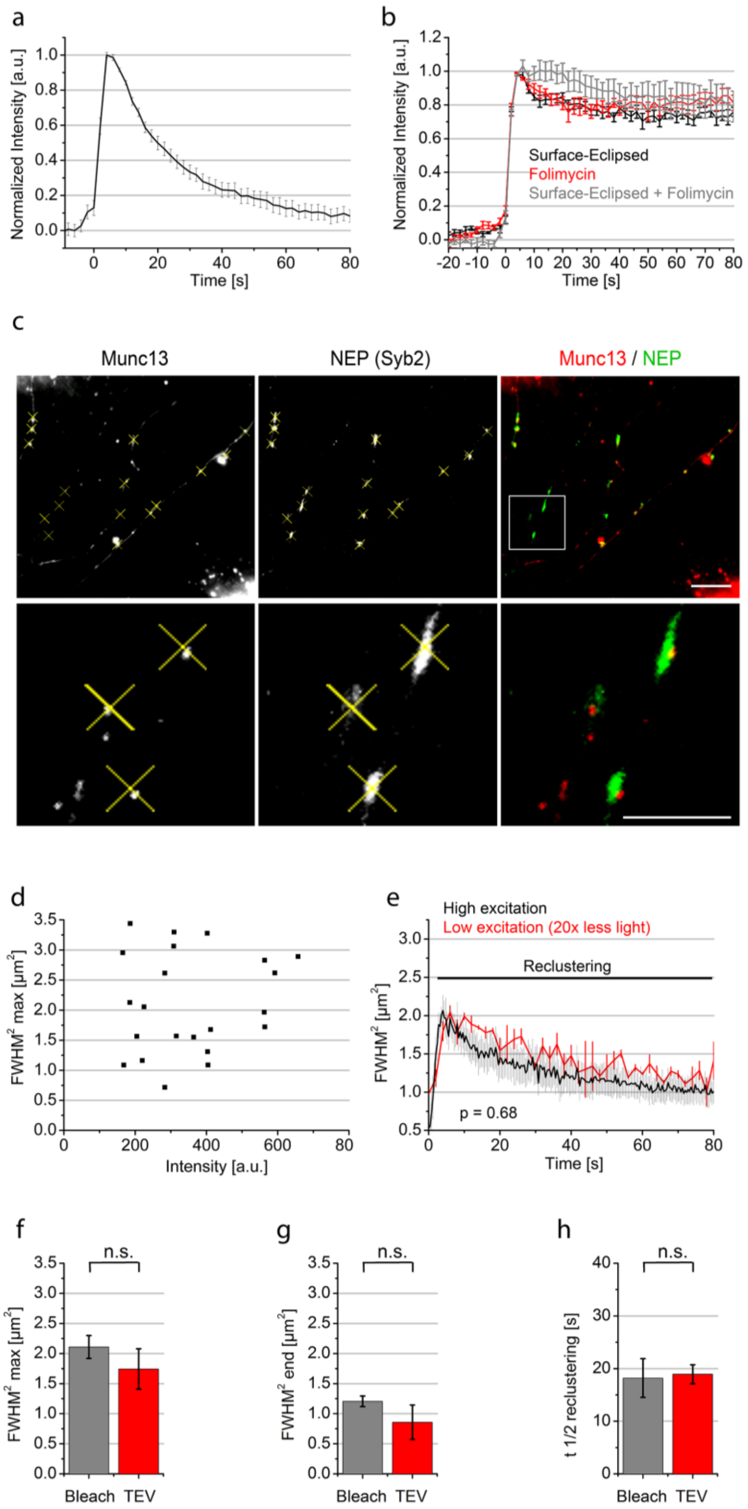

Figure S1: Syb2 spread, confinement and reclustering within presynaptic boutons.

(a,b) Hippocampal neurons expressing Syb2-pHluorin were stimulated in an electric field (40 AP, 20 Hz) and imaged as described. (a) Fluorescence intensity trace of pHluorin signals from non-eclipsed (non-photobleached) hippocampal neuron displaying exo- and endocytosis and reacidification of Syb2-pHluorin (mean ± s.e.m.; N = 3 independent experiments). (b) Surface-eclipsing by photobleaching, blocking reacidification (folimycin), or the combination of both treatments largely prevent the post-exocytic fluorescence decay indicating that the majority (≥ 80%) of newly exocytosed Syb2-pHluorin molecules remain at the neuronal surface under these conditions (mean ± s.e.m.; N = 7 neurons). The limited decay observed at least in part is due to axonal escape (see Fig. 1d). **(c)** Surface-eclipsed hippocampal neurons coexpressing Syb2-pHluorin and Munc13-mCherry were stimulated in an electric field (40 AP, 20 Hz) and imaged over time. The NEP was calculated as described in Fig. 1. Yellow crosses represent automatically detected synapse centers, which are passed to the Gaussian fit routine. Scale bar, 10 µm, zoom 5 µm. **(d)** Hippocampal neurons expressing Syb2-pHluorin were surface-eclipsed and stimulated in an electric field (40 AP, 20 Hz). The maximal spread ($FWHM^2$ max) is plotted as a function of Syb2-pHluorin expression level (synaptic pHluorin intensity before eclipsing of surface proteins). Each data point corresponds to one neuron. **(e)** $FWHM^2$ traces for time-lapse imaging of Syb2-pHluorin using high excitation (black, 2.5 Hz, no neutral density filter) or low excitation (red, 0.5 Hz, neutral density filter 4). Mean ± s.e.m.; high excitation N = 5, low excitation N = 2 individual experiments; Statistical significance was tested using a two-

way Anova test with repeated measures. **(f-h)** Neurons were surface-eclipsed by either photobleaching or enzymatic cleavage of Syb2-pHluorin (harboring a TEV cleavage site) by TEV protease. $FMWH^2$ of Gaussian fit shows no significant difference with respect to maximal spread (c), last time point (d), and (e) half-life $t_{1/2}$ of reclustering. Mean ± s.e.m.; independent experiments: Photobleach (N = 5), TEV cleavage (N = 3). Statistical analysis was done by two-tailed unpaired t-test.

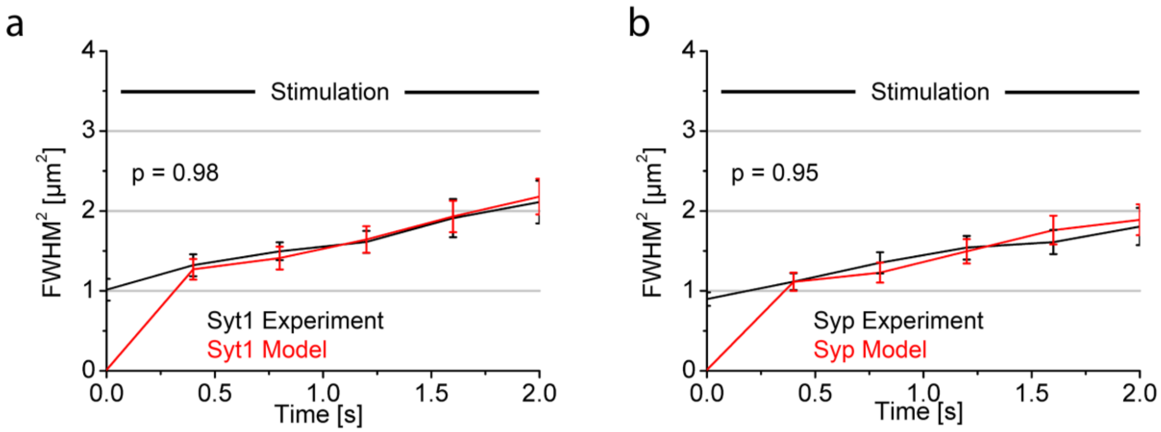

Figure S2: Newly exocytosed Syt1 and Syp undergo free diffusion.

Best-fit of model and experimentally determined $FMWH^2$ of surface-eclipsed Syt1- and Syp-pHluorin expressing hippocampal neurons stimulated by 40 APs at 20 Hz. Mean ± s.e.m.; independent experiments: Syt1 (N = 3), Syp (N = 3). Statistical significance was tested with a two-way Anova with repeated measures (time points 0.8-2s).

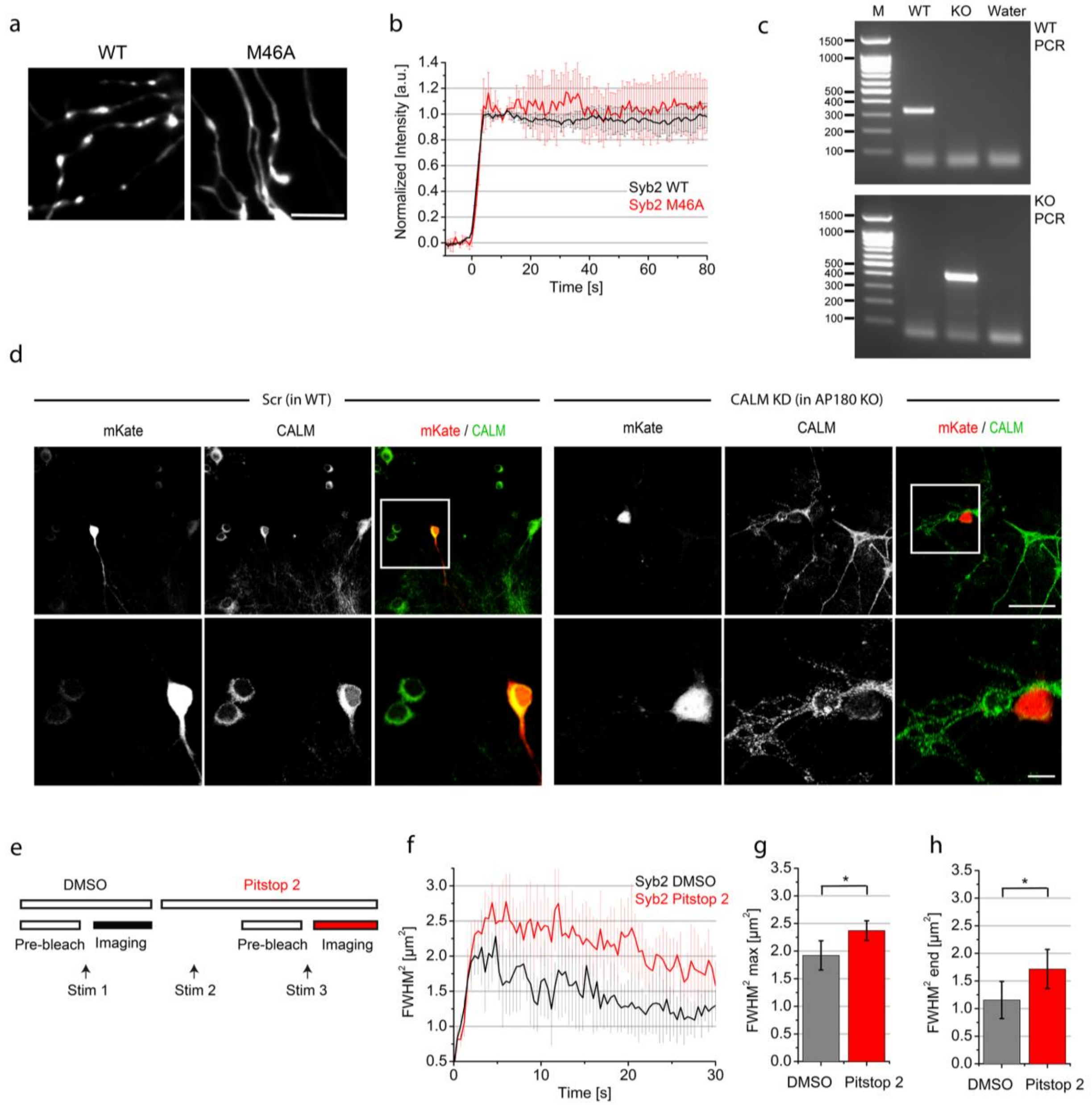

Figure S3: Spread and confinement are modulated by SV protein association with the endocytic machinery.

(a,b) Distribution of Syb2-pHluorin WT or M46A expressed in hippocampal neurons. Note the large axonal surface pool of Syb2 (M46A). Scale bar, 5µm (a). Neurons were surface-eclipsed, stimulated in an electric field (40 AP, 20 Hz) and imaged over time (b, 400 ms / frame). Intensity traces are from the data analyzed in Fig. 3b. **(c)** Verification of

AP180 knockout (KO) by PCR on genomic DNA using a primer set generating a 323 bp product in the presence of the WT allele (WT PCR) and a primer set generating a 401 bp product in presence of the KO allele (KO PCR). A reaction not containing genomic DNA (water) was run as control. **(d)** Levels of CALM in wild-type (WT) neurons expressing scrambled (scr) shRNA or in AP180 KO neurons expressing CALM-specific shRNA. mKate expressed from the same vector as the shRNA serves as transfection marker. Scale bar, 50 µm, zoom 10 µm. **(e-h)** Hippocampal neurons expressing Syb2-pHluorin were surface-eclipsed, treated with DMSO or Pitstop 2 and stimulated (40 AP, 20 Hz) as described in the scheme (e). $FWHM^2$ of the Gauss fit of the pHluorin signals over time show phases of spreading, confinement and reclustering (f) for both conditions. Maximal spread (g) and the last time point (h) were measured from data in (f). Mean ± s.e.m.; N = 3 individual experiments (b,f-h); Statistical significance was tested with a two-tailed paired t-test (g,h).

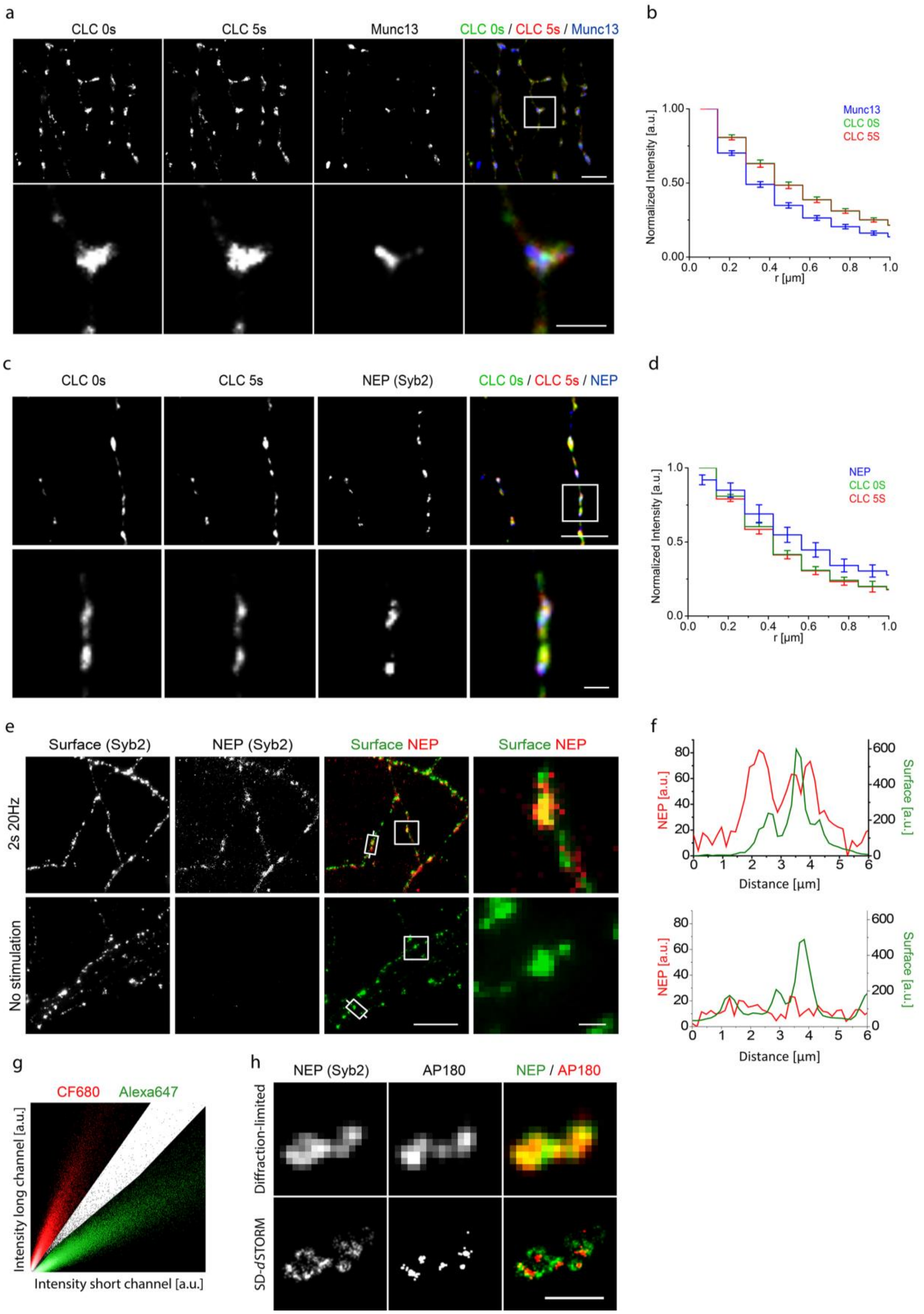

a
CLC 0s
CLC 5s
Munc13
CLC 0s / CLC 5s / Munc13
b
Munc13
CLC 0S
CLC 5S
Normalized Intensity [a.u.]
r [µm]
c
CLC 0s
CLC 5s
NEP (Syb2)
CLC 0s / CLC 5s / NEP
d
NEP
CLC 0S
CLC 5S
Normalized Intensity [a.u.]
r [µm]
e
Surface (Syb2)
NEP (Syb2)
Surface NEP
Surface NEP
2s 20Hz
No stimulation
f
NEP [a.u.]
Surface [a.u.]
Distance [µm]
NEP [a.u.]
Surface [a.u.]
Distance [µm]
g
CF680
Alexa647
Intensity long channel [a.u.]
Intensity short channel [a.u.]
h
NEP (Syb2)
AP180
NEP / AP180
Diffraction-limited
SD-dSTORM

Figure S4: Subsynaptic distribution of clathrin and newly exocytosed SV proteins.

(a,b) Surface-eclipsed hippocampal neurons coexpressing Munc13-mCherry and eGFP-clathrin light chain (CLC) were stimulated in an electric field (40 AP, 20 Hz) and imaged over time. (a) Munc13 before stimulation was overlaid with CLC before (0s) and after (5s) stimulation. Scale bar, 5 µm, zoom 2 µm. (b) Radial intensity profiles of Munc13 and CLC around maxima of Munc13 (mean ± s.e.m.; N = 8 neurons). **(c,d)** Surface-eclipsed hippocampal neurons expressing Syb2-pHluorin and mRFP-clathrin light chain (CLC) were stimulated in an electric field (40 AP, 20 Hz) and imaged over time. (c) NEP was calculated as described in Fig. 1 and overlaid with CLC before (0s) and after (5s) stimulation. Scale bar, 10 µm, zoom 1 µm. (d) Radial intensity profiles of NEP and CLC around maxima of NEP (mean ± s.e.m.; N = 2 independent experiments). **(e)** Hippocampal neurons expressing Syb2-Myc were stimulated with 40 APs at 20 Hz and stained as described in legend to Fig. 5. Newly exocytosed Syb2 only partially overlapped with surface-stranded Syb2 present on the neuronal surface prior to stimulation. Note the absence of newly exocytosed Syb2 without stimulation. Scale bar, 10 µm, zoom 1 µm. **(f)** Line plot of data shown in (e). **(g,h)** SD-*d*STORM imaging of newly exocytosed Syb2 (stimulation: 40 AP, 20 Hz) in hippocampal neurons expressing Syb2-c-myc. (g) Intensity space to assign colors for each single molecule localization in SD-*d*STORM imaging. Localizations were either assigned to the CF680 channel (black area, left), Alexa647 channel (black area, right) or not assigned (white area). (h) “Diffraction-limited” rendering and SD-*d*STORM images of AP180 and newly exocytosed Syb2. Scale bar, 1 µm.

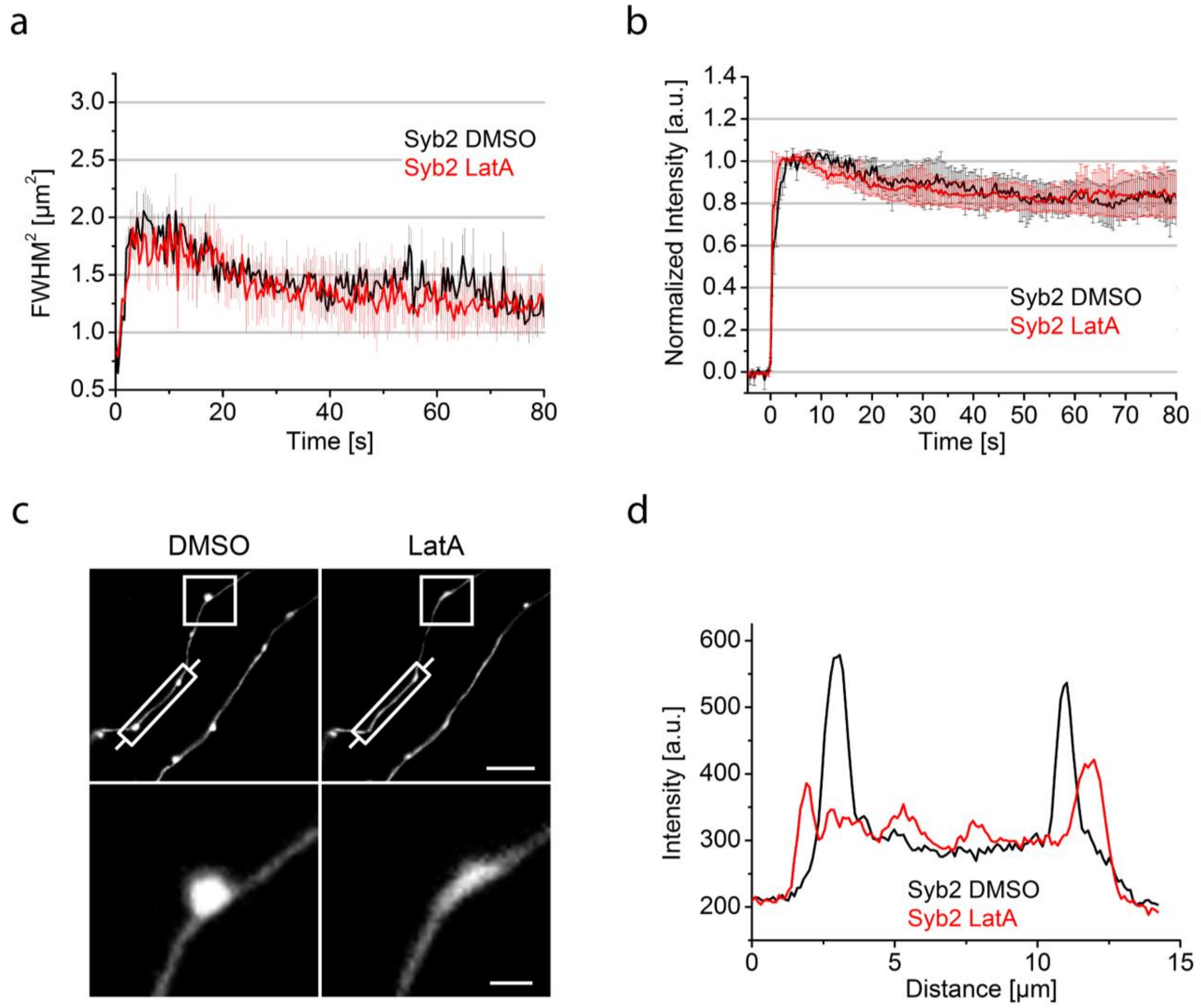

Figure S5: Treatment of neurons with the actin depolymerizing drug latrunculin A does not affect spreading, confinement, and reclustering of newly exocytosed SV proteins. (a,b) Hippocampal neurons expressing Syb2-pHluorin were surface-eclipsed, treated with DMSO or Latrunculin A and stimulated (40 AP, 20 Hz). (a) FWHM^2 of the Gaussian fit of the pHluorin signals over time shows no obvious differences in spreading, confinement, and reclustering. Mean ± s.e.m.; N = 3 individual experiments. (b) Fluorescence intensity traces of the data analyzed in (a). **(c,d)** Hippocampal neurons expressing LifeAct-eGFP were imaged in the presence of DMSO and after incubation

with Latrunculin A. (c) Representative images. Scale bar, 5 µm, zoom 1 µm. (d) Line plot of data shown in (c).
